# *Trypanosoma cruzi *(Chagas' disease agent) reduces HIV-1 replication in human placenta

**DOI:** 10.1186/1742-4690-5-53

**Published:** 2008-07-01

**Authors:** Guillermina Laura Dolcini, María Elisa Solana, Guadalupe Andreani, Ana María Celentano, Laura María Parodi, Ana María Donato, Natalia Elissondo, Stella Maris González Cappa, Luis David Giavedoni, Liliana Martínez Peralta

**Affiliations:** 1 National Reference Center for AIDS, Microbiology Department, School of Medicine, University of Buenos Aires, Buenos Aires, Argentina; 2 Laboratory of Parasitology, Microbiology Department, School of Medicine, University of Buenos Aires, Buenos Aires, Argentina; 3 Department of Virology and Immunology, Southwest National Primate Research Center (SNPRC), Southwest Foundation for Biomedical Research (SFBR), San Antonio, Texas, USA; 4 Endocrinology Service, Department of Clinical Biochemistry, José de San Martín Hospital, School of Pharmacy and Biochemistry, University of Buenos Aires, Buenos Aires, Argentina

## Abstract

**Background:**

Several factors determine the risk of HIV mother-to-child transmission (MTCT), such as coinfections in placentas from HIV-1 positive mothers with other pathogens. Chagas' disease is one of the most endemic zoonoses in Latin America, caused by the protozoan *Trypanosoma cruzi*. The purpose of the study was to determine whether *T. cruzi *modifies HIV infection of the placenta at the tissue or cellular level.

**Results:**

Simple and double infections were carried out on a placental histoculture system (chorionic villi isolated from term placentas from HIV and Chagas negative mothers) and on the choriocarcinoma BeWo cell line. Trypomastigotes of *T. cruzi *(VD lethal strain), either purified from mouse blood or from Vero cell cultures, 24 h-supernatants of blood and cellular trypomastigotes, and the VSV-G pseudotyped HIV-1 reporter virus were used for the coinfections. Viral transduction was evaluated by quantification of luciferase activity. Coinfection with whole trypomastigotes, either from mouse blood or from cell cultures, decreased viral pseudotype luciferase activity in placental histocultures. Similar results were obtained from BeWo cells. Supernatants of stimulated histocultures were used for the simultaneous determination of 29 cytokines and chemokines with the Luminex technology. In histocultures infected with trypomastigotes, as well as in coinfected tissues, IL-6, IL-8, IP-10 and MCP-1 production was significantly lower than in controls or HIV-1 transducted tissue. A similar decrease was observed in histocultures treated with 24 h-supernatants of blood trypomastigotes, but not in coinfected tissues.

**Conclusion:**

Our results demonstrated that the presence of an intracellular pathogen, such as *T. cruzi*, is able to impair HIV-1 transduction in an *in vitro *system of human placental histoculture. Direct effects of the parasite on cellular structures as well as on cellular/viral proteins essential for HIV-1 replication might influence viral transduction in this model. Nonetheless, additional mechanisms including modulation of cytokines/chemokines at placental level could not be excluded in the inhibition observed. Further experiments need to be conducted in order to elucidate the mechanism(s) involved in this phenomenon. Therefore, coinfection with *T. cruzi *may have a deleterious effect on HIV-1 transduction and thus could play an important role in viral outcome at the placental level.

## Background

Mother-to-child transmission (MTCT) of human immunodeficiency virus type 1 (HIV-1) occurs mainly when the newborn comes in contact with infected secretions of the mother during birth, though HIV-1 can also be transmitted through breastfeeding and *in utero *[1]. MTCT rates between 1–2% have been achieved after successful application of preventive therapies, mainly in industrialized countries [2-5]. However, studies performed in large cohorts with a follow-up of 8 years have shown that *in utero *transmission may still occur before therapy is initiated or effective [6]. Thus, this type of transmission seems to be a relevant way of MTCT even when efficient antiretroviral treatment and avoiding breastfeeding are being successfully performed.

The exact mechanisms by which the fetus acquires HIV-1 during pregnancy are not yet clear, even though the placenta is an efficient natural barrier that plays a role in the regulation of MTCT [7,8]. Soluble factors in the placental environment are part of this barrier. Indeed, several studies have suggested that cytokines and chemokines may be major regulators of transplacental transmission of HIV-1 [9-12]. A recent study demonstrated that placental explants from HIV-1 positive treated women secreted higher levels of leukemia inhibitory factor (LIF), interleukin (IL)-16, and regulated upon activation of normal T cells expressed and secreted (RANTES), soluble factors that inhibit HIV replication, and lower levels of TNF-α and IL-8, proinflammatory factors known as stimulators of viral replication [13].

Maternal viral load and immunological status are the main factors that determine the risk of HIV-MTCT [14,15]. Other risk factors are coinfections of the mother [16,17], an important issue since world regions with the highest prevalence of HIV-1 infection are also affected by other infections. Thus, HIV positive pregnant women are usually infected with other pathogens, and such placental coinfections may have consequences on MTCT of the pathogens. This is the case for HIV-1 infected pregnant women of sub-Saharan Africa coinfected with *Plasmodium falciparum*, who showed an increased peripheral and/or placental viral replication with more adverse birth outcomes than HIV uninfected women, particularly multigravida women [18]. Also noted, a shift in cytokine production towards a proinflammatory profile has been associated with *P. falciparum *placental infection [19,20], which could stimulate HIV-1 replication [21].

In Latin America, one of the most important endemic protozoonoses is Chagas' disease, caused by the protozoan parasite *Trypanosoma cruzi*. It extends from southern USA to southern South America. There are approximately 16–18 million infected people, representing the largest parasitic disease burden on the continent, with around 50,000 deaths per year and 100 million at risk of infection [22,23]. Largely considered as a rural entity, Chagas' disease has become an urban public health problem due to mass migration of rural inhabitants to big cities and an increase in poverty [24]. This "urbanization" of Chagas' disease facilitates coinfection in the most important areas for HIV prevalence: the City of Buenos Aires and surrounding areas. *T. cruzi *is mainly transmitted to humans by vectors such as blood-sucking bugs present in rural areas, but also by blood transfusion or congenital transmission. Due to the development of national programs for vector control and for the selection of blood donors, congenital transmission in women of child-bearing age still remains a pressing public health issue since *T. cruzi *could be transmitted to their newborn throughout the course of infection [23]. The rates of congenital transmission vary from 1% to 10%, according to geographic areas [25]. Such transmission takes place more frequently in the chronic stage of Chagas' disease, in endemic as well as in non-endemic areas, though its mechanisms have not been clearly defined [24,26]. In the case of *T. cruzi *infected mothers, no preventive treatment is possible during pregnancy due to the antiparasitic drugs' toxicity for the fetus [27]. Indeed, clinical management of these women differs greatly for HIV infected mothers.

Data from HIV-*T. cruzi *coinfected patients indicated reactivation of parasite infection with exacerbation of clinical signs and unusual clinical manifestations [28-31]. Even if no evidence exist focus on clinical features of coinfected mothers, MTCT of both pathogens with severe outcome for the children [32] and congenital transmission of *T. cruzi *without confirmation of HIV-1 MTCT [33] were reported. However, little is known about interaction of both pathogens on an *in vitro *cellular or *ex vivo *tissue model. Thus, the purpose of the study was to determine whether coinfection with *T. cruzi *and HIV-1 at the tissue or cellular level modifies HIV-1 infection.

## Results

### Tissue viability and responsiveness to stimuli

**Figure 1:**
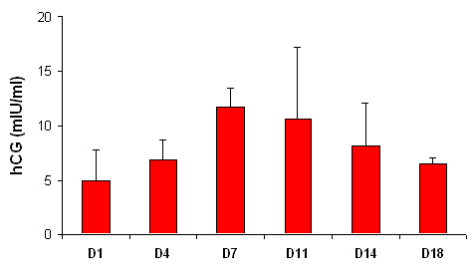
**Production of hCG in the culture medium of placental histocultures**. Chorionic villi were placed on 1.5 cm^2 ^collagen sponge gels at medium-air interface into the wells of 6-well plates, 9 blocks per collagen sponge and per well. Production of hCG was measured in histoculture supernatants every 3 to 4 days from day 1 to day 18 or 21 by the chemiluminescence method. Placental histocultures were maintained in 5% CO_2 _atmosphere/95% air at 37°C. Results represent mean ± SD of duplicates and are representative of 3 independent experiments.

Viability of the placental histocultures throughout the culture period was evaluated by quantifying total hCG production in histoculture supernatants every 3 to 4 days from day 1 to day 18 or 21. The maximum level of total hCG was observed at day 4 or 7. A decrease was observed between day 7 and day 11 of culture, and the minimum level was reached after day 18. These levels are comparable to those obtained in histocultures from previously reported term placentas [34]. Kinetics of hCG production are shown in Figure 1.

After the set-up of the histoculture system and before the start of the infection protocols, the tissue response to an external stimulus such as LPS was evaluated. Placental histocultures were stimulated with 0.1 and 10 μg/ml LPS at day 0, 3 and 6 for 24 h before supernatant collection. Placental histocultures showed a response to this stimulus in a dose-dependent manner secreting high amounts of TNF-α at all the times tested (data not shown).

### Tissue function is not modified by pseudotyped virus transduction and or parasite infection

**Figure 2:**
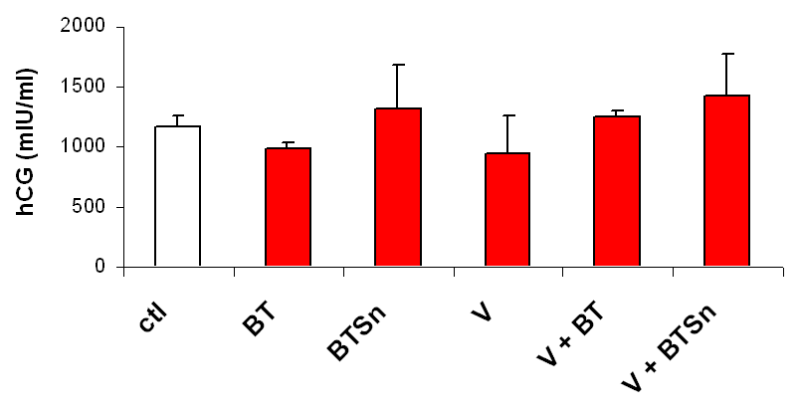
**Tissue functionality after pseudotyped virus transduction and/or parasite infection**. Placental villi were dissected and immediately transducted overnight with VSV-G pseudotyped HIV-1 (V) (100 ng p24/placental block) alone or in the presence of blood trypomastigotes (BT) (10^6 ^parasites/placental block) or 24 h-supernatant of BT (BTSn). After infection or coinfection, tissue functionality of placental histocultures was analyzed by measuring total hCG secretion levels in histoculture supernatants by the chemiluminescence method at day 4 post-infection or coinfection. Results represent mean ± SD of duplicates and are representative of 5 independent experiments.

After viral transduction with or without parasite infection, tissue function of placental histocultures was analyzed by measuring total hCG secretion levels in histoculture supernatants from each experiment. As shown in Figure 2, neither viral nor parasite treatment significantly modified hCG secretion, indicating that the outcome of infection was not due to direct cytotoxicity of the inocula for the histocultures.

### *T. cruzi *trypomastigotes and 24 h-supernatants of trypomastigotes decrease HIV-1 replication in BeWo cells

**Figure 3:**
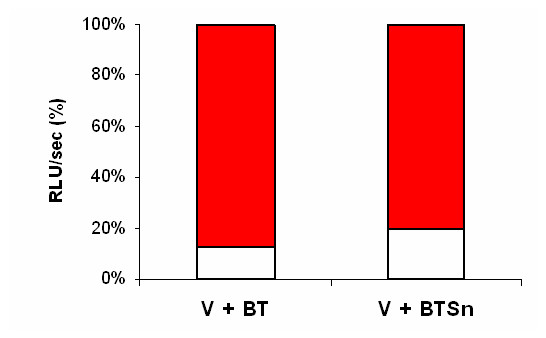
**Effect of blood *T. cruzi *trypomastigotes and 24 h-supernatant of trypomastigotes on HIV-1 replication in BeWo cells**. The human choriocarcinoma BeWo cell line was transducted overnight with VSV-G pseudotyped HIV-1 (V) (100 ng p24/2 × 10^4 ^cells per well) alone or in the presence of blood trypomastigotes (BT) (2 × 10^5 ^parasites/2 × 10^4^cells per well) or 24 h-supernatant of BT (BTSn). Cells were lysed and luciferase activity as an indicator of viral replication was read from cell lysates at day 4 post-infection or coinfection. Results are expressed as relative light units per second (RLU/sec), presented as a percentage relative to VSV-G. The histogram in red corresponds to the % of infection with VSV-G (= 100%) and the histogram in white corresponds to the % of infection in the presence of BT. Results are representative of 3 independent experiments.

The effect of blood trypomastigotes (BT) on HIV-1 replication was assessed on BeWo cells, a model of early trophoblast cells which are the first placental layer in direct contact with maternal blood. Previous data indicated that the HIV-1 R5 (BaL) or X4 (HXB2) pseudotyped reporter virus did not replicate in BeWo cells [35], thus we used only VSV-G pseudotyped HIV-1 reporter virus. Cells were incubated with BT and/or pseudotyped virus, and transduction of the luciferase reporter gene as an indicator of viral replication was evaluated at the end of the experiment. As shown in Figure 3 (left bar), viral replication was decreased by BT (-86%, *p *< 0.005).

As trypomastigotes shed several soluble factors [36], we wanted to determine whether the trypomastigote supernatant could interfere with HIV-1 replicative cycle, or if an active *T. cruzi *infection was necessary to achieve the previously described effect. Thus, BeWo cells were incubated with 24 h-supernatants from BT (BTSn) and VSV-G pseudotype virus. Similar effect on luciferase activity as in the case of BT was observed for BTSn (-76%, *p *< 0.005) (Figure 3, right bar).

### *T. cruzi *trypomastigotes and 24 h-supernatants of trypomastigotes decrease HIV-1 replication in placental histocultures

**Figure 4:**
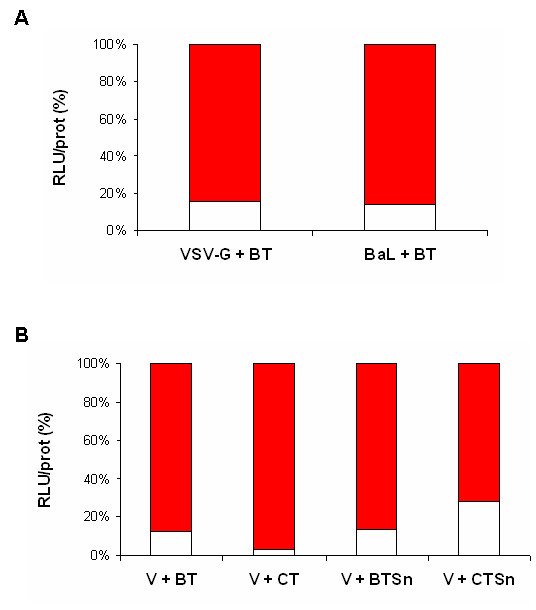
**Effect of blood and culture *T. cruzi *trypomastigotes and 24 h-supernatants of trypomastigotes on HIV-1 replication in placental histocultures**. **A: **Placental villi were transducted overnight with BaL (B) (250 ng p24/placental block) or VSV-G pseudotyped HIV-1 (V) (100 ng p24/placental block) alone or in the presence of blood trypomastigotes (BT) (10^6 ^parasites/placental block). Fragments were homogenized and luciferase activity as an indicator of viral replication was read from tissue lysate at day 4 post-infection or coinfection. Results are expressed as relative light units per second (RLU/sec), presented as a percentage relative to B or V and were normalized in each sample by total protein concentration (RLU/prot). The histogram in red corresponds to the % of infection with BaL or VSV-G (= 100%) and the histogram in white corresponds to the % of infection in the presence of BT. **B: **Placental villi were transducted overnight with VSV-G pseudotyped HIV-1 (V) (100 ng p24/placental block) alone or in the presence of blood trypomastigotes (BT) or cell trypomastigotes (CT) (10^6 ^parasites/placental block), or 24 h-supernatants of BT (BTSn) or 24 h-supernatants of CT (CTSn). Fragments were homogenized and luciferase activity as an indicator of viral replication was read from tissue lysate at day 4 post-infection or coinfection. Results are expressed as relative light units per second (RLU/sec), presented as a percentage relative to V and were normalized in each sample by total protein concentration (RLU/prot). The histogram in red corresponds to the % of infection with VSV-G (= 100%) and the histogram in white corresponds to the % of infection in the presence of BT, CT, BTSn or CTSn (*p *< 0.001). Results are represented as a mean of 5 independent experiments.

Transduction with pseudotyped virus harboring VSV-G, HIV-1 R5 (BaL) or HIV-1 X4 (HXB2) envelope protein were performed on placental histocultures, with or without infections with BT. Results were normalized in each sample by total protein concentration. When placental histocultures were transducted with HXB2 pseudotyped virus, no luciferase activity was detected (data not shown). When BaL pseudotyped virus was used, even at higher doses than VSV-G pseudotyped virus, levels of luciferase activity were lower. However, for both pseudotyped virus luciferase activities were significantly decreased in coinfection with BT (mean ± SD; -90.98% ± 5.83, *p *< 0.001 for VSV-G and -94% ± 5.02, *p *< 0.001 for BaL) (Figure 4A).

Purification of BT might carry other components from mouse blood, mainly white cells and platelets, which could interfere with HIV-1 replication. Thus, similar experiments were performed using trypomastigotes purified from Vero cell culture supernatants (CT). Similarly, live CT significantly decreased virus-driven luciferase activity in placental histocultures when they were coinfected with VSV-G pseudotyped HIV-1 reporter virus (mean ± SD; -97.36% ± 0.98, *p *< 0.001) (Figure 4B).

Additionally, placental explants were incubated with 24 h-supernatants from either BT (BTSn) or CT (CTSn) and VSV-G pseudotype virus. A similar effect on luciferase activity as in the case of BT was observed for BTSn (mean of diminution ± SD; -81.48% ± 8.15, *p *< 0.001), while CTSn also decreased luciferase activity although at a lower degree (-61.21% ± 1.94, *p *< 0.001) (Figure 4B).

### Effect of coinfection on soluble factor secretion

**Figure 5:**
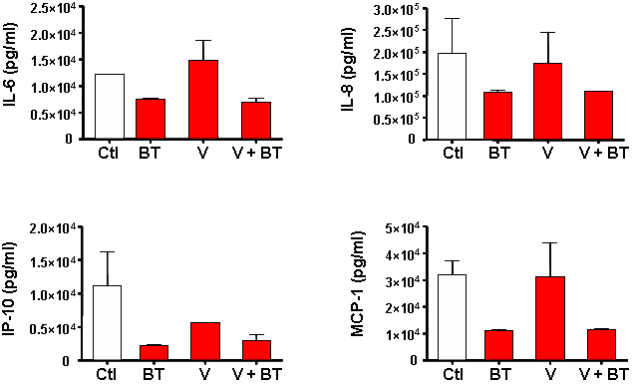
**Effect of blood *T. cruzi *trypomastigotes on cytokine/chemokine secretion in placental histocultures**. Placental villi were transducted overnight with VSV-G pseudotyped HIV-1 (V) (100 ng p24/placental block) alone or in the presence of blood trypomastigotes (BT) (10^6 ^parasites/placental block). Histoculture supernatants were collected after infection or coinfection, diluted with 10% FCS in RPMI and used for the simultaneous determination of cytokine/chemokine production with Luminex technology. Results displayed correspond to IL-6 (*p *< 0.01), IL-8 (*p *< 0.05), IP10 (*p *< 0.01) and MCP-1 (*p *< 0.02). Results are expressed as the mean ± SD and are representative of 5 independent experiments.

In an attempt to determine whether changes in the placental microenvironment due to parasite-viral interaction are responsible for inhibiting HIV replication, cytokine/chemokine secretion was measured in histoculture supernatants at day 1 and at day 4 post-infection or coinfection. Results for day 1 presented in Figure 5 demonstrate that *T. cruzi *acts as a potent inhibitor of IL-6 (*p *< 0.01), IL-8 (*p *< 0.05), IP10 (*p *< 0.01) and MCP-1 (*p *< 0.02), while no significant changes were observed in RANTES, MIP-1α, MIP-1β, G-CSF, GRO-α, GM-CSF, IFN-γ, IL-10, IL-13, IL-1β, IL-2, IL-4 and IL-5 production (data not shown). The effect of HIV-*T. cruzi *interaction on cytokine/chemokine secretion seems to be parasite-driven since their levels correlated with those induced by the parasite alone.

**Figure 6:**
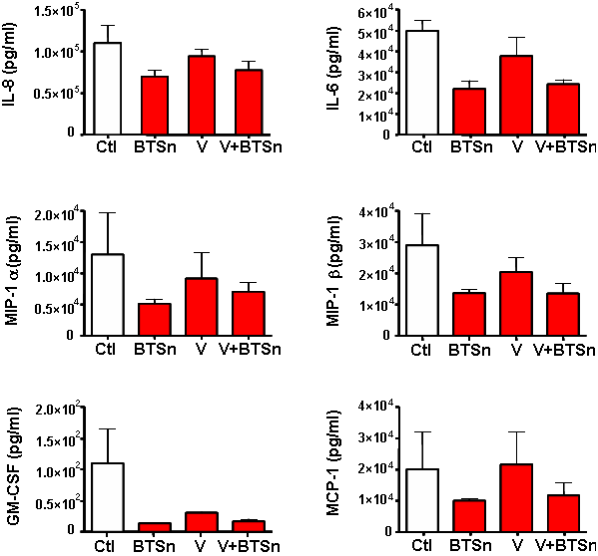
**Effect of 24 h-supernatants of blood *T. cruzi *trypomastigotes on cytokine/chemokine secretion in placental histocultures**. Placental villi were transducted overnight with VSV-G pseudotyped HIV-1 (V) (100 ng p24/placental block) alone or in the presence of 24 h-supernatants of blood trypomastigotes (BTSn) (10^6 ^parasites/placental block). Histoculture supernatants were collected after infection or coinfection, diluted with 10% FCS in RPMI and used for the simultaneous determination of cytokine/chemokine production with Luminex technology. Results displayed correspond to IL-6 (*p *< 0.04), IL-8 (*p *< 0.05), MIP-1α (*p *< 0.05), MIP-1β (*p *< 0.05), GM-CSF (*p *< 0.05) and MCP-1 (*p *< 0.01) (pg/ml). Results are expressed as the mean ± SD and are representative of 5 independent experiments.

When placental histocultures were treated with BTSn, significant decreases only in IL-6 (*p *< 0.04), IL-8 (*p *< 0.05), MCP-1 (*p *< 0.01), GM-CSF (*p *< 0.05), MIP-1α (*p *< 0.05), and MIP-1β (*p *< 0.05) secretion were detected in histoculture supernatants collected at day 1 post-infection or coinfection (Figure 6). Surprisingly, this diminution was detected only in BTSn treated histocultures but no changes were observed in HIV-BTSn treated samples.

In all experiments, the differences seen at day 1 were no longer seen at day 4 post-infection or coinfection.

## Discussion

As a result of the significant burden of the HIV pandemics in resource-poor regions, a number of potential epidemiological, biological, and clinical interactions between HIV and other tropical pathogens gained relevance and need to be studied. The interactions between HIV and tropical infectious agents are complex. Each pathogen has the potential to alter the epidemiology, natural history, and/or response to therapy of the other pathogens [37]; therefore, it is unpredictable to establish the outcome of such coinfections. In Latin America, one of the most significant endemic protozoonoses is Chagas' disease, and several clinical studies from HIV-*T. cruzi *coinfected patients have been reported [28-31]. MTCT is one way of transmission shared by both pathogens. The exact mechanisms involved in MTCT of both pathogens are not clear. Hence, the study of their interaction at the placental level is critical for designing strategies that abolish MTCT.

In our *in vitro *culture system of term placental histocultures, as well as in the trophoblast cell line BeWo, we demonstrate that acute coinfection with *T. cruzi *and HIV-1 pseudotyped virus decreases HIV-1 replication. This is the first report about interaction of these pathogens at the placental level.

In order to validate our placenta *in vitro *model, we evaluated the viability and responsiveness to stimuli of the histocultures. As previously described [34], micro-explant villi of term placentas were morphologically viable until day 7 or 11 with no significant alterations (data not shown) and total hCG secretions peaked at day 7 or 11. Additionally, they were able to react to external stimuli such as LPS, secreting a great amount of TNF-α, as previously described [38]. Altogether, these results provide evidence that placental villi are intact and remain viable and functional until at least day 7 of culture.

For BeWo cells and placental histoculture infection, a pseudotyped Vesicular Stomatitis Virus G/HIV-1 was used. This pseudotyped virus, due to its amphotropic nature, is able to infect any cell type, regardless of receptor and coreceptor surface expression [39]. When R5-Env pseudotyped HIV-1 was used on placental histocultures, coinfection with trypomastigotes also abolished HIV-1 replication almost completely (Fig. 4A). Previous studies have demonstrated that X4-Env HIV-1 pseudotype viruses do not infect human term placental chorionic villi and that a higher dose of R5-Env HIV-1 pseudotypes, compared to viruses pseudotyped with VSV-G, is necessary to observe an infection of placental tissue [21]. Similarly, previous studies have shown that malignantly transformed human cell lines of the trophoblast lineage are resistant to cell-free HIV-1 pseudotypes bearing the R5 and X4 envelopes, and that this resistance was bypassed when HIV-1 envelopes were substituted by the VSV-G protein [35]. Thus, subsequent experiments of our study on placental histocultures as well as on BeWo cell line were performed only with VSV-G pseudotyped virus. Although we do not address the effects of *T. cruzi *on the binding of HIV-1 to their natural receptors, our experiments are valid in studying the effects of the parasite on viral replication.

A great impairment of HIV-1 replication was observed in coinfection with viable *T. cruzi *trypomastigotes purified from mouse blood (BT). Moreover, when other source of viable trypomastigotes was used, such as those grown in cell culture (CT), the same effect on HIV-1 replication was observed. In all cases, hCG secretion was measured in histoculture supernatants and no significant differences were observed between control, viral infection, or treatment with trypomastigotes. These results indicate that placental tissue remains viable and that parasite impairment of HIV-1 replication was not associated with direct cell toxicity caused by *T. cruzi*. Previous data indicate that the parasite induces rearrangement of cortical cytoskeleton of syncytiotrophoblast with actin microfilament depletion during human placental invasion [40].

Considering that after entering the cell, the HIV-1 virion interacts initially with actin filaments which assist binding to microtubules and transport to the nuclear periphery [41], modifications in trophoblast cytoskeleton might impair viral replication at an early phase in the case of active *T.cruzi *invasion. However, the inhibition of HIV replication seems to be caused not only by viable trypomastigotes but also by soluble factors shed by the parasite, either from BT or CT.

Taking into account that *T. cruzi *sheds several proteases (cysteine, serine, threonine and metalloproteinases) that participate in host cell invasion [36], those molecules could interfere with some critical structures inside the cytosol of host cells required for viral genome retrotranscription and transfer to the nucleus. Indeed, this is allowed by the reverse transcription complex that later becomes the preintegration complex, and both complexes include not only viral RNA or DNA and several accessory viral proteins, but also cellular proteins [42], which are necessary for efficient reverse transcription of HIV-1 [43]. Disruption of these complexes by exogenous enzymes or alteration of protein interactions can lead to an impaired HIV-1 replication [44]. We might hypothesize that this is the case when *T. cruzi *proteases are present.

Since the *T cruzi *is a complex intracellular organism that has a great impact on host cell structure and also on its metabolism, we decided to evaluate whether the parasite or its soluble products are able to modify the placental environment. In fact, many soluble factors, including cytokines and hormones, with regulatory activities are essential for establishing and maintaining pregnancy [45,46]. HIV-1 and antiretroviral treatment in pregnant women have an impact on the pattern of placental soluble factors [13,38]. On the other hand, little is known about the changes in human fetal-maternal interface in *T. cruzi *infection. In histocultures infected with trypomastigotes and in coinfected tissue, IL-6, IL-8, IP-10 and MCP-1 production was significantly lower than in controls or HIV-1 infected tissues. Certainly, most of these chemokines are an important driving-force for CD8^+ ^T-cell recruitment, which plays a significant role in the control of acute *T. cruzi *infection [47]. Concerning HIV-1, many reports describe the role of cytokines/chemokines on its replication. Among them, IL-6 is a well-known cytokine with up-regulating activity on HIV replication [48]. Moreover, MCP-1 and IP-10 were associated with an increase in leukocyte density and cerebrospinal fluid viral load [49-51].

Indeed, IP-10, as well as IL-8 may stimulate HIV-1 replication in different cell types [52,53], although mechanisms are not clearly defined. In our system, a diminished secretion of those stimulatory cytokines/chemokines was observed at day 1 post-infection whenever the parasite was present. These transitory changes in the placental environment might contribute to HIV-1 replication impairment.

Parallel studies have also been conducted on another cellular system for the study of HIV/*T. cruzi *interaction as one of the main cell targets for both pathogens, the monocyte-derived macrophages, and similar inhibition of viral replication was observed at different levels of HIV-1 replication cycle (Andreani G. *at al*., manuscript in preparation). Thus, related mechanisms by which *T. cruzi *impair HIV-1 replication seem to be involved in different *in vitro *systems.

## Conclusion

Our results demonstrate that the presence of an intracellular pathogen such as *T. cruzi *is able to impair HIV-1 transduction in an *in vitro *system of human placental histocultures. Direct effects of the parasite on cellular structures as well as on cellular/viral proteins essential for HIV-1 replication might influence viral transduction in this model. Nonetheless, additional mechanisms including modulation of cytokines/chemokines at placental level could not be excluded in the inhibition observed. Further experiments need to be conducted in order to clarify the mechanism(s) involved in this phenomenon.

In summary, coinfection with *T. cruzi *may have a deleterious effect on HIV-1 transduction and thus could play an important role in viral outcome at the placental level.

## Methods

### Histocultures of chorionic villi from term placentas

Term placentas were obtained after programmed cesarean section at the Obstetrics Unit of the Fernández and Ramos Mejía Hospitals in the city of Buenos Aires, in accordance with Argentinean ethics guidelines. This study was approved by the Ethics Committee from the School of Medicine, University of Buenos Aires. Histocultures of chorionic villi were performed as previously described [34] with slight modifications. Briefly, placental villi were isolated, washed extensively with RPMI 1640 (CellGro, USA) and dissected into 2–3 mm blocks. After infection or coinfection, chorionic villi were placed on 1.5 cm^2 ^collagen sponge gels (Espongostan, Johnson & Johnson, USA) at medium-air interface into the wells of 6- or 12-well plates (Greiner, Germany) with 3 or 2 ml media per well respectively, at 9 tissue blocks per collagen sponge and per well. Histoculture medium was RPMI 1640 (CellGro) supplemented with 15% heat-inactivated fetal calf serum (FCS, PAA-Bioser, Argentina), 1% penicillin-streptomycin, 0.1% gentamicin, 1% L-glutamine, 1% non-essential amino acids, 1% sodium pyruvate; (Gibco BRL Ltd., USA). Placental histocultures were maintained in 5% CO_2 _atmosphere/95% air at 37°C. Each experimental point means a duplicate histoculture well.

### Evaluation of histoculture viability and response to stimuli

Viability of histocultures was monitored by detection of total hCG secretion determined with a chemiluminescence method (Immulite 1000, detection limit 1.1 mIU/ml, Siemens Medical Solutions Diagnostics, USA) in supernatants from both histoculture system setup protocols (from day 1 to day 18) and in infection protocols (day 4 post-infection or coinfection). Tissue responses to LPS were analyzed by incubating tissue fragments with 0.1 and 10 μg/ml LPS (from *E. coli *serotype 055:B5, Sigma-Aldrich, USA) at day 0, 3 and 6 of histoculture. After 24 h, levels of Tumor Necrosis Factor-alpha (TNF-α) secretion were quantified by ELISA (Peprotech, Mexico) in histoculture supernatants.

### Trophoblast cell line

The human choriocarcinoma BeWo cell line [54], used as a model for early trophoblast cells [55-57], was obtained from the American Type Culture Collection (ATCC # CCL98, Rockville, Md.). These cells were maintained in Dulbecco's Modified Eagle Medium (DMEM, CellGro) containing 25 mM glucose, supplemented with 20% heat-inactivated FCS, 20 mM glutamine, 50 IU/ml penicillin and 50 μg/ml streptomycin, in 5% CO_2 _atmosphere/95% air at 37°C.

### Pseudotyped viruses

Luciferase reporter viruses were produced as previously described [35] by transiently cotransfecting (SuperFect; Qiagen, Germany) 293T cells with the proviral pNL-Luc-E^-^R^+ ^vector [58], which lack the *env *gene and has the firefly luciferase gene inserted into the *nef *gene, and the expression vector pCMV harboring the gene coding for either the VSV-G envelope protein or the HIV-1 R5 (BaL) envelope protein [59], or the expression vector pSV harboring the gene coding for HIV-1 X4 (HXB2) envelope protein [60]. Supernatants from 293T cells were harvested 72 h after transfection and p24*gag *levels were measured using a commercial ELISA kit (Murex, UK).

### *T. cruzi *purification and supernatant preparation

*T. cruzi *VD strain (isolated from a case of congenital Chagas' disease, lethal for mice, lineage II) was used [61]. This subpopulation was maintained by serial passages in 21-day old CF1 mice. Either bloodstream forms (BT) or tissue culture-derived (CT) trypomastigotes were employed for the coinfection assays. BT were collected from blood of *T. cruzi *infected mice at the peak of parasitemia by cardiac puncture. To enrich blood supernatants with BT, the centrifuged blood was incubated for 1 h at 37°C and the supernatant was collected. Thus, BT were pelleted by centrifugation for 30 min at 10,000 × g, counted in a Neubauer hematocytometer and diluted to 10^7 ^BT/ml in BeWo medium or to 4 × 10^7 ^BT/ml in histoculture medium for further use in coinfection assays. In order to obtain CT, Vero cell monolayers were allowed to interact with BT in a parasite/cell ratio of 5:1 for 24 h. CT harvested from the second passage in Vero monolayers were pelleted by centrifugation for 30 min at 10,000 × g, counted in a Neubauer hematocytometer and diluted to 4 × 10^7 ^CT/ml in histoculture medium for further use in coinfection assays.

In order to obtain parasite supernatants, 10^7 ^BT diluted in BeWo medium or 4 × 10^7 ^BT or CT diluted in histoculture medium were incubated for 24 h at 37°C in 5% CO_2_. To remove parasites and cellular debris, both parasite suspensions were pelleted as described above and the supernatants were filtered through a 0.22 μm pore-size filter. Filtrated aliquots were stored at -80°C until use for coinfection assays.

### Infection of trophoblast cells

BeWo cells were seeded in 96-well plates (2 × 10^4^cells per well) 24 h before infection and then incubated with VSV-G pseudotyped HIV-1 (100 ng of p24 per well) and 2 × 10^5 ^BT or 24 h-supernatant of BT (BTSn) overnight at 37°C in 5% CO_2_. Controls included transduction with only the pseudotyped virus or Δ*env *pseudotype, infection with parasites or treatment with parasite supernatants, and also mock infected cells with culture medium. After culture for an additional 72 h, 100 μl of luciferase lysis buffer (Promega) per well was added and luciferase activity as an indicator of viral replication was measured in 10 μl of lysate with a luminometer (Veritas), using the commercially available substrate; data were expressed as RLU/sec.

### Infection of placental histocultures

After dissection, 18 blocks of placental villi were placed in 24-well plates and transducted overnight with BaL (250 ng p24/placental block), HXB2 (250 ng p24/placental block) or VSV-G pseudotyped HIV-1 (100 ng p24/placental block) and infected with BT or CT (10^6 ^parasites/placental block), or parasite supernatants. Controls included transduction with only the pseudotyped virus or Δ*env *pseudotype, infection with parasites or treatment with parasite supernatants, and also mock infected histocultures with culture medium. The following day, placental blocks were washed 6 times in 6-well plates with PBS 1× (Gibco BRL Ltd.) using a cell strainer (BD Biosciences, USA), placed on collagen sponges and cultured as described above for an additional 72 h. Protocols of overnight infection and then supernatant collection were also performed.

For further cytokine/chemokines quantification, at the end of each experiment, supernatants were collected, clarified by centrifugation at 1,000 × g for 10 minutes, filtered (0,22 μm), aliquoted and stored at -80°C. Placental fragments were collected and preserved at -80°C until homogenization.

For luciferase activity quantification, placental fragments were homogenized in 500 μl of luciferase lysis buffer (Promega, USA) with an Ultra-turrax homogenizer (IKA, USA). Luciferase activity was measured at day 4 post-infection or coinfection in 20 μl of lysate with a luminometer (Veritas, USA), using a commercially available substrate (Dual-Luciferase Reporter Assay System, Promega), and expressed as relative light units (RLU). Results were normalized to total protein concentration measured on lysates from each sample using a Micro BCA™ Protein Assay Kit (Pierce, USA). Final data were expressed as RLU/μg prot (RLU/prot).

### Quantification of the protein secretion of soluble factors in placental histocultures

Supernatants of stimulated histocultures were diluted with 10% FCS in RPMI and used for the simultaneous determination of 29 cytokines and chemokines with Luminex technology as previously described [62,63]. The coated bead/biotinylated antibody combinations used were: G-CSF (LINCOplex human G-CSF, Linco Research, St. Charles. MO), GM-CSF (Beadlyte human GM-CSF, Upstate USA, Charlottesville, VA), GRO-α (Beadlyte human GRO-α, Upstate), IFN-α (anti-human IFN-α clones MMHA-11 and MMHA-2, PBL Biomedical Laboratories, Piscataway, NJ), IFN-γ (Beadlyte primate IFN-γ, Upstate), IL-1β (Monkey IL-1β ELISPOT reagents, U-Cytech), IL-1Ra (Fluorokine MAP human IL-1Ra/IL-1F3, R&D System, Minneapolis, MN), IL-2 (Beadlyte primate IL-2, Upstate), IL-4 (LINCOplex human IL-4, Linco), IL-5 (LINCOplex human IL-5, Linco), IL-6 (LINCOplex human IL-6, Linco), IL-7 (anti-human IL-7 clone 7417 and polyclonal anti-human IL-7, R&D), IL-8 (Beadlyte human IL-8, Upstate), IL-9 (Beadlyte human IL-9, Upstate), IL-10 (anti-human IL-10 clones BN-10 and QS-10, Cell Sciences Inc., Canton, MA), IL12(p40) (anti-human IL-12 clones IL-12I and IL-12II, Mabtech Inc., Mariemont, OH), IL-12(p70) (anti-human IL-12 p70 clone 20C2, Endogen, and IL-12II, Mabtech), IL-13 (Beadlyte human IL-13, Upstate), IL-15 (anti-human IL-15 clone 34505 and polyclonal anti-human IL-15, R&D), IL-17 (Human IL-17, Biosource International, Camarillo, CA), IL-18 (anti-human IL-18 clones 125-2H and 159-12B, MBL International, Woburn, MA), IP-10 (anti-human IP-10 clone 33036 and polyclonal anti-human IP-10, R&D), MCP-1 (Human MCP-1, Biosource), MIP-1α (Human MIP-1α, Biosource), MIP-1β (Human MIP-1β, Biosource), RANTES (Beadlyte human RANTES, Upstate), sCD40L (Fluorokine MAP human sCD40L, R&D System), TNF-α (Beadlyte human TNF-α, Upstate), and TNF-β (Beadlyte human TNF-β, Upstate). Cytokine concentrations were determined using human cytokines (Upstate) as standards and the Masterplex QT software from Mirabio.

### Statistical Analysis

Results of luciferase activity and cytokine/chemokine production from each group (infected or coinfected tissue/cells) are presented as mean ± SD. Comparison of their distributions between 2 groups for luciferase activity was performed by a *t*-Student test and distribution between more than 2 groups for cytokine/chemokine expression was performed by a non-parametric Friedman test and a post-test Dunns, using the Graph Pad Prism 4 software.

## Competing interests

The authors declare that they have no competing interests.

## Authors' contributions

GLD was responsible for the design, testing and writing of the manuscript. GLD and GA were responsible for viral preparation and for all the coinfection experiments in the *in vitro *placental model and BeWo cells. MES and AMC were responsible for the isolation, culture and characterization of the parasites, and contributed to writing the manuscript. SMGC contributed to the design of the experiments and discussion of the manuscript. LMP^1 ^and LDG performed and interpreted the cytokine measurements. AMD and NE performed all the hormonal determinations. LMP^2 ^was responsible for the design and writing of the manuscript. All authors read and approved the final manuscript.

^1 ^Laura María Parodi

^2 ^Liliana Martinez Peralta
